# The Development and Psychometric Validation of the Fainareti Screening Tool for Perinatal Mental Health in Greek Pregnant Women

**DOI:** 10.3390/clinpract15020037

**Published:** 2025-02-14

**Authors:** Maria Dagla, Irina Mrvoljak-Theodoropoulou, Vassilis Daglas, Evangelia Antoniou, Eleni Rigoutsou, Alexandros Papatrechas, Calliope Dagla, Eleni Tsolaridou, Despoina Karagianni

**Affiliations:** 1 Day Center for the Care of the Mental Health of Women (Perinatal Mental Health Disorders), Non-Profit Organization “FAINARETI”, 17121 Athens, Greece; irina.mrvoljak@psych.uoa.gr (I.M.-T.); v.daglas@fainareti.gr (V.D.); lilanton@uniwa.gr (E.A.); e.rigoutsou@fainareti.gr (E.R.); a.papatrechas@fainareti.gr (A.P.);; 2 Laboratory of Midwifery Care During Antenatal and Post Natal Period-Breastfeeding, Department of Midwifery, School of Health & Care Sciences, University of West Attica, 12243 Athens, Greece; 3 Department of Psychology, National & Kapodistrian University of Greece, 11528 Athens, Greece

**Keywords:** anxiety, pregnancy, screening tool, perinatal mental health, Fainareti mental health screening tool

## Abstract

**Background/Objectives**: It would be helpful for primary healthcare professionals to have access to a brief, general screening tool allowing them to detect patients suffering from major mental illness. This also holds for organizations and institutions at which pregnant women ask for support during the perinatal period. An evaluation of the psychometric properties, validity, and reliability of the Fainareti mental health screening tool was carried out in Greek women in this study. **Methods**: The study participants consisted of 518 women retrospectively followed from pregnancy to their first year postpartum as part of a health intervention at the Day Center for Women’s Mental Health Care (Perinatal Mental Health Disorders), operated by the non-profit organization Fainareti. Alongside the newly developed screening tool, this study utilized the Perinatal Anxiety Screening Scale (PASS), the Patient Health Questionnaire (PHQ-9), and the Edinburgh Postnatal Depression Scale (EPDS). **Results**: The assessment of the tool’s internal reliability included computing two separate internal consistency indices, with both indicating its significant level of reliability. The correlation analysis between the tool and the scales included in this study demonstrated the tool’s strong convergent validity, while factor analyses confirmed its satisfactory construct validity. **Conclusions**: Overall, these findings suggest that the one-factor Fainareti mental health screening tool is suitable for initial assessments of the mental health of Greek women.

## 1. Introduction

During pregnancy, one undergoes considerable physiological and psychological changes, increasing one’s likelihood of experiencing distressing feelings, also referred to as perinatal mood and anxiety disorders (PMADs) [1]. A study conducted by Kendig et al. 2017 [2] found that 13% to 21% of pregnant women suffer from PMADs, which are characterized by anxiety and depression. It has been suggested that healthcare providers should routinely screen for PMADs in all prenatal care patients with more consistent follow-up [3] because PMADs go undetected in 50 percent of those with these conditions [4]. Major depressive disorders and perinatal depression share many symptoms; PMADs, however, are triggered by pregnancy [5]. In addition, anxiety disorders and depression co-occur during the perinatal period [6].

The rate of postpartum depression is 7 to 15% [7]. This is a significant percentage suffering from this condition considering that a mother having postpartum depression hinders the bond between her and her child, which adversely affects the development of her child’s brain. Several studies have found it to negatively impact cognitive development, behavioral inhibition, and emotional maladjustment, with a risk of psychiatric disorders in offspring over time [6,8]. In addition to postpartum depression, postpartum anxiety has also been linked to negative psychological outcomes in offspring, such as increased behavioral and emotional problems [9,10]. Specifically, several adverse birth outcomes have been linked to PMADs, including preterm births, intrauterine growth [8,11], decreased bonding between mothers and infants, and delayed cognitive and emotional development [12,13].

In general, experiencing depressive and anxiety disorders while pregnant appears to significantly increase the likelihood of developing postpartum depression [14,15,16] and, as mentioned above, can contribute to negative outcomes for the child [17]. However, depression and anxiety have often been identified as significantly underdiagnosed in primary care settings, with their actual prevalence rates being 5 to 10 times higher than those commonly reported [18].

Experiencing an anxiety disorder can impede a woman’s capacity to find enjoyment in life and engage in self-care. If this kind of disorder emerges during the perinatal period, encompassing pregnancy and/or the initial year postpartum, it can alter the way a woman experiences her pregnancy and influence her interactions with and care for her child [19]. Also, common mental disorders (CMDs), characterized by symptoms of depression and anxiety, pose a significant concern during the perinatal period due to their debilitating impact on maternal functioning, as well as social, economic, and personal fulfilment. Just as much as PMADs, CMDs can have adverse effects on the health and development of infants and children [20].

Globally, in approximately 80% of women experiencing CMDs during the perinatal period, they go unrecognized or untreated [21]. In Greece, antenatal care is provided by obstetricians and midwives working in primary care settings (public or private) and hospitals, while pediatricians also participate in postpartum care. Although these professionals are expected to identify pathological mental health symptoms in pregnant women and new mothers during the routine provision of perinatal care, psychological assessments are often not routinely integrated into antenatal or perinatal care. Therefore, it is essential to conduct screenings for these disorders during both the antenatal and postpartum periods.

Furthermore, in developed nations, the prevalence of maternal depression varies between 7% and 15% [8], while in low- and middle-income countries (LMICs), its prevalence, as measured using both screening and diagnostic tools, can be as high as 20–26% [22]. Beyond depression, evidence indicates that anxiety is a frequent occurrence during pregnancy and might be even more prevalent than depression. A systematic review by Brunton and his colleagues reported global prevalence rates of anxiety during pregnancy ranging from as low as 18% to as high as 60% [23]. Suicidal ideation and behavior have also increasingly been reported during the perinatal period, with prevalence rates ranging between 6% and 18% [24,25,26,27].

Professionals widely agree that the recognition of women potentially experiencing antenatal depression or anxiety by trained healthcare personnel is considered good clinical practice [28,29]. This process of identification can be enhanced by structured approaches, including the use of standard identification questions [30,31] or dedicated assessment tools [28]. The Perinatal Anxiety Screening Scale (PASS) serves as an anxiety screening tool for both pregnant and postpartum women. It proves beneficial throughout the perinatal period for evaluating a variety of anxiety symptoms [32,33]. The standard depression screening instruments for both postpartum and pregnant individuals encompass the Patient Health Questionnaire (PHQ-9) and the Edinburgh Postnatal Depression Scale (EPDS) [34,35,36].

To bridge the gap between the lengthy EPDS and the concise Whooley Questions, a six-item mental health screening tool was created for pregnant women in Greece. It is administered on first contact with a pregnant woman, in person or via the phone, at the Day Center for the Care of the Mental Health of Women, run by the non-profit organization Fainareti. This tool is designed to identify pregnant women exhibiting symptoms of common mental disorders (CMDs) and suicidal ideation. The objective of the current research is to determine the validity of the Fainareti six-item mental health screening tool, as compared with that of three commonly used instruments, the PASS, PHQ-9, and EPDS, and to assess its psychometric characteristics.

## 2. Materials and Methods

### 2.1. Sample

This study relied on data collected from 518 women who gave birth in Athens, Greece, from January 2016 to January 2019. The average age of these women was *M* = 35.90 ± 4.12, with their ages ranging from 26 to 47 years. All of the participants were actively engaged in an advanced psychosocial health intervention conducted at the Day Center for the Care of the Mental Health of Women (Perinatal Mental Health Disorders). Only women who attended the Day Center’s complete program (from pregnancy to the 1st year postpartum) participated in this study. This center, operated by the non-profit organization Fainareti, stands as the only Day Center of its kind in the nation. Oversight and funding for this Day Center, a pivotal community facility, are provided by the Mental Health Department of the Greek Ministry of Health. Every participant in this study provided informed oral and written consent to the analysis of their data for research purposes, with awareness of their right to withdraw their consent at any time. Participation in the use of the screening tool took place at the Day Center, typically between the 18th and 22nd week of gestation. Approval for this study was granted by the Research Ethics Committee of the non-profit organization Fainareti (Ref. Number 77/03.07.19).

### 2.2. Instrumentation

The *Perinatal Anxiety Screening Scale (PASS)* and the *Patient Health Questionnaire (PHQ-9)*, utilized in this current research, underwent back-translation procedures. The scale was initially translated from English to Greek by translators without prior knowledge of the research project. Subsequently, it was independently translated back into English by two additional translators.

The *Perinatal Anxiety Screening Scale—PASS* [32]—is a robust and reliable 31-item screening tool designed to assess significant anxiety in pregnant and postpartum women. Each item employs a 4-item Likert scale ranging from 0 (“not at all”) to 3 (“almost always”). The total score is the sum of the individual scores, with higher totals indicating greater anxiety. The scores range from 0 to 93, and the clinical cutoff for anxiety is set at 26. By addressing specific anxiety symptoms in perinatal women across four domains, it distinguishes between groups at high and low risk of anxiety disorders. The domains form subscales, including (1) General Worry and Specific Fears (10 items); (2) Perfectionism, Control, and Trauma (8 items); (3) Social Anxiety (5 items); and (4) Acute Anxiety and Adjustment (8 items). The PASS demonstrates good convergent validity, as evidenced by its significant correlations with the EPDS’s anxiety subscale and overall EPDS scores. The scale exhibits excellent overall reliability, with a Cronbach’s alpha of 0.96. In the present study, Cronbach’s alpha reached 0.94, supporting its reliability.

The *Edinburgh Postnatal Depression Scale—EPDS* [37]—is a widely utilized screening tool for both antepartum and postpartum depression. Consisting of 10 items, it employs a 4-item Likert scale, where 0 represents the lowest grade and 3 the highest. The final score, ranging from 0 to 30 points, is derived by summing up the scores for all of the items. A total score of 12 or higher is considered a potential indicator of depression. The scale has undergone translation and validation for the Greek population by two distinct research groups [38,39], demonstrating robust overall internal consistency. In the validation study by Leonardou et al. [38], the Cronbach’s alpha for the total scale was 0.90, while in the study by Vivilaki et al. [39], it was 0.80. In the present study, the alpha coefficient was 0.88.

*The Patient Health Questionnaire—PHQ-9* [40]—serves as a screening scale designed to assess the presence and severity of depressive symptoms. Comprising the nine symptoms outlined in the DSM-IV Major Depressive Episode (MDE) criterion A, these items are structured into an adjectival scale format that measures the presence of symptoms over the previous two weeks (“not at all”, “several days”, “more than half the days”, and “nearly every day”). The scores, ranging from 0 to 3, are summed up to provide a total score between 0 and 27. The PHQ-9 can be self- or hetero-administered and is utilized algorithmically for a probable diagnosis of MDE or as a continuous measure with the cut-off points at 5, 10, 15, and 20 representing the levels of depressive symptoms (mild, moderate, moderately severe, and severe). The scale demonstrates satisfactory internal consistency, with a Cronbach’s alpha value ranging from 0.86 to 0.89. In the present study, the alpha coefficient was 0.87.

*The Fainareti mental health screening tool* was designed to assess various aspects of mental health, using a combination of established questions from previous research and an additional question developed by Fainareti professionals (psychologists and midwives). It consists of six questions in total, focusing on the symptoms of depression and anxiety, the need for professional help, and suicidal ideation. Two questions assessing depression were adapted from Whoolley et al. [41]. The first question asks, “During the past month, have you often been bothered by feeling down, depressed, or hopeless?” The second question asks, “During the past month, have you often been bothered by little interest or pleasure in doing things?” One question, taken from Arroll et al. [42], asks about the individual’s perception of the need for mental health support: “Do you feel a need to get in touch with a mental health professional?” Two questions regarding anxiety are based on the guidelines from the National Institute for Health and Care Excellence [43]. The first question asks, “During the past month, have you felt stressed, anxious, or pushed to your limits?” The second question asks, “During the past month, have you had trouble stopping or controlling your anxiety?” Finally, the Fainareti professionals added one question to assess suicidal ideation: “During the past month, have you thought about suicide?” If a respondent answers positively to any of these six questions, they are considered “positive” in terms of mental health concerns. This tool incorporates both evidence-based items from existing research and a question specifically developed by Fainareti professionals to provide a comprehensive mental health screening.

### 2.3. Statistical Analysis

The statistical analysis of the Fainareti mental health screening tool involved several key steps to assess its reliability and validity. Internal reliability was evaluated using Cronbach’s alpha and Guttman’s split-half coefficient, with values greater than 0.70 considered satisfactory. Inter-item correlations were also examined to ensure homogeneity among the scale items.

An exploratory factor analysis (EFA) was initially applied to identifying the underlying factor structure of the tool. The Principal Component extraction method was used with Varimax axis rotation and Kaiser normalization, and the Kaiser–Guttman criterion was applied using eigenvalues greater than one. The Kaiser–Meyer–Olkin (KMO) criterion and Bartlett’s test were used to confirm the suitability of the sample for the factor analysis.

A confirmatory factor analysis (CFA) was then conducted to test the structure identified using the EFA. This included testing an independence model, a default model, and a modified model with error covariances to achieve a good model fit. The chi-square, degrees of freedom, *p*-value, RMSEA, RMR, and AIC indices and the GFI, CFI, and TLI were used to evaluate the model’s goodness-of-fit.

Convergent validity was assessed using Spearman’s rank correlation coefficient to correlate the scores of the Fainareti tool with those of the PASS, the PHQ-9, and the EPDS.

## 3. Results

### 3.1. Descriptive Statistics and Reliability

The descriptive statistics and reliability indices of the scale are presented in Table 1. Internal consistency metrics were used to assess the Fainareti screening tool’s internal reliability (i.e., Cronbach’s alpha and Guttman’s split-half). The inter-item correlations were all statistically significant, ranging from 0.171 to 0.481, showing good item-to-scale homogeneity.

Both internal consistency coefficients were required to have values greater than 0.70 [44]. As shown in Table 1, Cronbach’s α coefficient for the screening tool was satisfactory (0.73). Additionally, Guttman’s split-half coefficient was similar, displaying the good internal consistency (0.72) of the present sample. The positively skewed data showed a low level of negative symptoms among the participants.

### 3.2. The Psychometric Properties of the Fainareti Mental Health Screening Tool

Initially, the EFA was applied to the Fainareti screening tool; it consists of six items. A CFA was conducted to additionally test the structure. Following the Kaiser–Guttman criterion of factor extraction for eigenvalues greater than one, a single factor was extracted that met this criterion. The analysis was performed using the Principal Component extraction method with Varimax axis rotation and Kaiser normalization (Table 2).

According to the Kaiser–Meyer–Olkin criterion (KMO = 0.77), the sample was suitable for a further analysis, as was the table of the interrelationships of the six questions, according to Bartlett’s criterion (χ^2^ = 319.28, *df* = 19, *p* < 0.001). The communalities for most items were above 0.30 as a threshold point. Since the correlation matrix and the factor loadings indicated no further irregularities in the items, no items were excluded from the analysis. One factor emerged to which 42.44% of the variance was attributed.

### 3.3. Structure Confirmation

**Figure 1:**
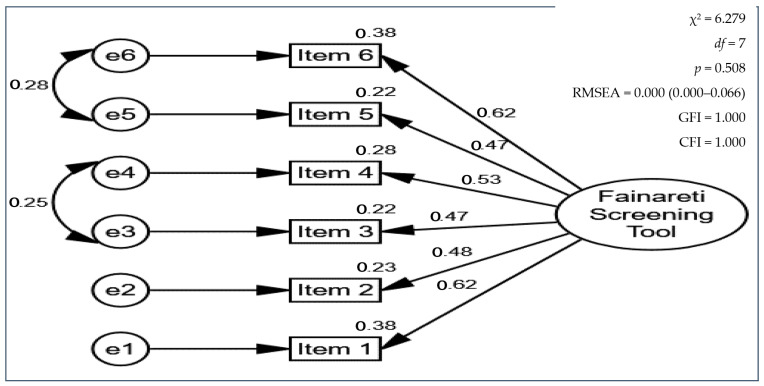
Confirmatory factor analysis for the Fainareti screening tool.

The CFA was employed in the following stage (Table 3 and Figure 1) since it cannot be expected to confirm the findings of an exploratory factor analysis in a different sample or population if it cannot be applied accordingly to the same data [45]. However, the EFA was employed mainly to present the items, while the focus was on the CFA.

The unifactorial model without modification indices had a satisfactory goodness of fit but with a high RMSEA. After some modifications to the model in the form of allowing two error covariances (specifically e3–e4 and e5–e6, as seen in Figure 1), the data for the model were fully confirmed. Since a CFA can include error covariances that indicate that two measures covary due to factors other than a shared factor’s influence, such as method effects, we opted to allow these error covariances to differ to result in the best-fitting model [46]. The chi-square index changed to become statistically nonsignificant, and the GFI, TLI, and CFI reached the highest possible values, while the RMSEA, RMR, AIC, and χ^2^/*df* declined.

### 3.4. Factor Validity

The assessment of convergent validity employed Spearman’s rank correlation coefficient, considering the skewness of the scale mentioned. In the context of the present study, the relationships between the Fainareti mental health screening tool and the PHQ-9, the EPDS, and the PASS were explored to measure the Fainareti mental health screening tool’s validity. The findings revealed substantial positive correlations with robust values: 0.425 with the PASS, 0.505 with the PHQ-9, and 0.531 with the EPDS. This indicates a significant positive correlation between the Fainareti mental health screening tool and each scale, demonstrating the outstanding convergent validity of the tool within the sample used in this study.

## 4. Discussion

The primary objective of this study was to ascertain the validity of the six-item mental health screening tool developed by the non-profit organization Fainareti and to evaluate its psychometric characteristics. To assess the tool’s internal consistency and homogeneity, various measures, including Cronbach’s alpha, Guttman’s split-half coefficient, and the item–total correlations, were calculated. The construct validity was examined using an EFA, which identified a one-factor scale within the study population. A CFA was also conducted to validate the identified one-factor model. Additionally, this study investigated the convergent validity by employing Spearman’s correlation coefficient. This involved comparing the total scores of the Fainareti mental health screening tool with those of the PASS, the PHQ-9, and the EPDS.

Τhese findings affirm the robust factor structure of the Fainareti mental health screening tool within the specific sample utilized in this study. The analyses’ robustness, as indicated by the Cronbach’s alpha (0.73), Guttman’s split-half coefficient (0.72), and inter-item correlation (ranging from 0.171 to 0.481), establishes the tool’s consistency, reliability, and acceptable homogeneity. The scale’s validity was also statistically significant, showing convergent validity between 0.425 and 0.531. This implies a substantial positive correlation between the Fainareti mental health screening tool and the PASS, the PHQ-9, and the EPDS in the perinatal period. Additionally, empirical evidence from this study supports the construct validity of the Fainareti mental health screening tool, revealing a one-factor structure with consistent EFA factor loadings aligning with the CFA model.

Several questionnaires have been developed to assist primary care providers in identifying mental health issues [47,48,49,50], but many practitioners find them too time-consuming and cumbersome to use routinely [51,52]. Taking an example symptom of depression, as defined by the Diagnostic and Statistical Manual of Mental Disorders-IV [53], major depressive episodes are characterized by either a depressed mood or a loss of interest or pleasure in most activities for at least two weeks. In primary care, the Primary Care Evaluation of Mental Disorders Procedure (PRIME-MD) involves completing a 27-item screening questionnaire and conducting a follow-up interview with a clinician. Among the questions in the questionnaire are two Whoolley Questions related to depressed mood and anhedonia which are also part of the Fainareti mental health screening tool: (1) “During the past month, have you often been bothered by feeling down, depressed, or hopeless?” and (2) “During the past month, have you often been bothered by little interest or pleasure in doing things?” [54]. Comparing a diagnosis of major depression via a phone interview with a positive response to one of these two questions, the PRIME-MD study reported that a positive answer to one of these two questions was 86% sensitive and 75% specific [54]. It has been suggested that patients that answer yes to one of these questions are evaluated in terms of other symptoms such as fatigue, restlessness, suicidal ideation, guilt, poor concentration, and changes in sleep or appetite. However, the brevity of the six-question tool, which also contains questions about anxiety and a question about suicidal thoughts, makes it the most suitable instrument for routine use. The Fainareti one-factor mental health screening tool could significantly enhance university and protocol training by providing a streamlined, effective method for the early detection of mental health issues, fostering the better integration of mental health care into routine healthcare settings.

Despite the promising results of this study, there are several limitations that should be acknowledged. First, the sample was drawn from a specific group of women in Athens, Greece, and may not be fully representative of perinatal women in different cultural, socioeconomic, or healthcare contexts, limiting the generalizability of the findings. Additionally, since the Fainareti screening tool relies on self-reported responses, there is the potential for bias, including social desirability bias or a reluctance to disclose sensitive mental health information. While this study compared the Fainareti tool to established scales like the PHQ-9, EPDS, and PASS, further comparisons with a broader range of screening tools would offer more context regarding its relative effectiveness. Furthermore, the cross-sectional nature of this study meant that the tool’s performance was evaluated at only one point in time, and longitudinal studies would be needed to assess its ability to track changes in mental health over time. Finally, this study’s focus on the perinatal population also restricts the tool’s applicability to other groups, and further validation would be needed to determine its effectiveness in diverse populations.

## 5. Conclusions

In conclusion, our findings confirm that the Fainareti mental health screening tool is valid in identifying mental health problems in pregnancy, probably in settings where face-to-face questions are possible during general health discussions. If the respondent responds positively to some of the questions, a further clinical assessment may be required. In addition to indicating to pregnant women that their mental and physical health is addressed by this service, the questions on mental health can be asked easily and quickly at routine planned appointments. These questions, in the context of a supportive open discussion, offer the opportunity for women’s responses concerning their psychosocial circumstances to be discussed as well. Time-confined clinical practice may benefit from this tool, so we recommend its incorporation. It would be helpful to conduct a similar study validating the questions when asked by mental health professionals who are knowledgeable about these issues.

## Figures and Tables

**Table 1:** Descriptive statistics and reliability coefficients for Fainareti mental health screening tool.

(*N* = 518)	*M*	*SD*	Min	Max	Alpha	Split-Half	*N*
Total Score	1.33	1.59	0.00	6.00	0.73	0.72	6

**Table 2:** Exploratory factor analysis for the Fainareti mental health screening tool.

	Factor
1. During the past month, have you often been bothered by feeling down, depressed or hopeless?	0.696
2. During the past month, have you often been bothered by little interest or pleasure in doing things?	0.580
3. During the past month, have you felt stressed, anxious or pushed to your limits?	0.526
4. During the past month, have you had trouble stopping or controlling your anxiety?	0.623
5. During the past month, have you thought about suicide?	0.780
6. Do you feel a need to get in touch with a mental health professional?	0.661

**Table 3:** Confirmatory factor analysis of the Fainareti mental health screening tool.

	*M1*	*M2*	*M3*
χ^2^	322.287	36.792	6.279
*df*	15	9	7
*p*	*p* < 0.001	*p* < 0.001	*p* = 0.508
χ^2^/*df*	21.459	4.108	0.897
RMSEA [90% CI]	0.260 [0.235, 0.285]	0.101 [0.068, 0.136]	0.000 [0.000, 0.066]
RMR	0.044	0.009	0.003
GFI	0.666	0.961	1.00
CFI	-	0.910	1.00
TLI	-	0.849	1.00
AIC	334.287	60.792	34.279
Δ*χ*^2^		^M1–M2^ 285.495	^M1–M3^ 316.008 ^M2–M3^ 30.513
Δ*df*		^M1–M2^ 6	^M1–M3^ 8 ^M2–M3^ 2
*p*		^M1–M2^ *p* < 0.001	^M1–M3^ *p* < 0.001 ^M2–M3^ *p* < 0.001

Notes: M1—independence model; M2—default model; M3—modified model.

## Data Availability

The original contributions presented in this study are included in the article. Further inquiries can be directed to the corresponding author.
